# Roles of microRNAs and RNA-Binding Proteins in the Regulation of Colorectal Cancer Stem Cells

**DOI:** 10.3390/cancers9100143

**Published:** 2017-10-24

**Authors:** Junko Mukohyama, Yohei Shimono, Hironobu Minami, Yoshihiro Kakeji, Akira Suzuki

**Affiliations:** 1 Division of Molecular and Cellular Biology, Kobe University Graduate School of Medicine, Kobe, Hyogo 650-0017, Japan; junkom@med.kobe-u.ac.jp (J.M.); suzuki@med.kobe-u.ac.jp (A.S.); 2 Division of Gastrointestinal Surgery, Kobe University Graduate School of Medicine, Kobe, Hyogo 650-0017, Japan; kakeji@med.kobe-u.ac.jp; 3 Department of Pathology and Cell Biology, Department of Medicine (Division of Digestive and Liver Diseases) and Herbert Irving Comprehensive Cancer Center (HICCC), Columbia University, New York, NY 10032, USA; 4 Division of Medical Oncology/Hematology, Kobe University Graduate School of Medicine, Kobe, Hyogo 6500017, Japan; hminami@med.kobe-u.ac.jp

**Keywords:** cancer stem cell, colorectal cancer, microRNA, RNA binding protein

## Abstract

Colorectal cancer stem cells (CSCs) are responsible for the initiation, progression and metastasis of human colorectal cancers, and have been characterized by the expression of cell surface markers, such as CD44, CD133, CD166 and LGR5. MicroRNAs (miRNAs) are differentially expressed between CSCs and non-tumorigenic cancer cells, and play important roles in the maintenance and regulation of stem cell properties of CSCs. RNA binding proteins (RBPs) are emerging epigenetic regulators of various RNA processing events, such as splicing, localization, stabilization and translation, and can regulate various types of stem cells. In this review, we summarize current evidences on the roles of miRNA and RBPs in the regulation of colorectal CSCs. Understanding the epigenetic regulation of human colorectal CSCs will help to develop biomarkers for colorectal cancers and to identify targets for CSC-targeting therapies.

## 1. Introduction

Colorectal cancer is the third most commonly diagnosed cancer in males and the second in females, with an estimated 1.4 million cases worldwide [1]. Current therapies are not fully capable of curing colorectal cancers in advanced stages. The difficulty to cure advanced colorectal cancers is at least partly attributed to the presence of a small population of highly-tumorigenic cancer cells termed cancer stem cells (CSCs) which are responsible for the initiation, progression and metastasis of colorectal cancer [2,3,4,5,6].

Initiation and progression of colorectal cancers are driven by genetic and epigenetic modifications. Indeed, stepwise accumulation of genetic mutations is first presented in colorectal cancers [7]. In addition, it is evident that epigenetic regulation, such as histone modification, promoter methylation and microRNA (miRNA) regulation, is important for cancer development and progression.

miRNAs are non-coding RNAs with fewer than 25 nucleotides, and function as epigenetic regulators of protein expression. Each miRNA can regulate translation of mRNAs, based on its capacity to recognize a unique target sequence (seed sequence) in the 3′ untranslated region (3′ UTR) of target mRNAs [8]. miRNAs regulate a variety of cell functions, including cell proliferation, stem cell maintenance and differentiation. We previously identified miRNAs, such as miR-200 families and miR-142, can target key elements of the self-renewal and multi-lineage differentiation pathways in human breast CSCs and normal mammary stem/progenitor cells by analysis of the surgical specimens of human breast cancer patients [9,10]. However, few studies have focused on miRNAs specifically involved in the regulation of CSCs in human colorectal cancer tissues.

RNA-binding proteins (RBPs) associate with RNAs to form ribonucleoprotein complexes and control the biogenesis, activity and stability of RNAs and miRNAs [11]. For example, pre-mRNA processing reactions, including splicing, editing and polyadenylation, are mediated by RBPs and trans-acting RNAs; and RBPs, such as HuR and Dead end 1 (Dnd1), bind to the 3′ UTR of target mRNA and suppress the miRNA-mediated translational suppression [12]. Existence of interplay between RBPs and RNAs is crucial for various biological processes, such as development and maintenance of stem cell phenotypes.

In this review, we focus on human colorectal CSCs, especially those directly isolated from the surgical specimens of colorectal cancer patients, and describe current knowledge and future perspectives on the important roles of miRNA and RBPs in the epigenetic regulation of CSC properties in human colorectal cancers.

## 2. Human Colorectal Cancer Stem Cells

Human colorectal cancer tissues contain distinct cell populations, including a CSC population, whose transcriptional identities mirror those of the distinct cellular lineages of normal colorectal epithelium. Isolation of colorectal CSCs and/or normal intestinal stem/progenitor cells from patient specimens is accomplished based on the expression of one or multiple cell surface markers, such as CD44, CD133, CD166, and leucine-rich repeat-containing G-protein coupled receptor 5 (LGR5) [4,5,6,13]. Other markers include EphB2 [14], aldehyde dehydrogenase (ALDH) [15], CD66c [16] and CD44v6 [17].

### 2.1. Shared Molecular Properties between Colorectal CSCs and Normal Intestinal Stem Cells

Human tissues maintain their architecture over time through a tightly regulated process of renovation. Colorectal epithelial cells are continuously replaced every few days. This process is driven by self-renewing stem cells at the base of colorectal crypts, which constantly support the production of a progeny that progressively migrates toward the intestinal lumen as it undergoes multi-lineage differentiation into a variety of mature cell types [18]. Epithelial cell properties are mainly studied in small intestine; however, there are several differences in crypt structure and cell composition between the epithelium of small and large intestine. The colorectal crypt does not protrude to form villi at the mucosal surface, and it does not contain Paneth cells and +4 cells [2].

Colorectal CSCs share a part of the stem cell properties with normal intestinal stem/progenitor cells, and cellular composition shows some similarities between colorectal tumors and the normal colorectal epithelium. Dalerba and colleagues investigated the cellular composition of the normal colonic epithelium and benign and malignant colon tumors, by applying single cell gene expression analysis [19]. They revealed that the cell populations characterized by distinct gene signatures, such as immature progenitor (*LGR*5^+^/*ASCL*2^+^) and goblet-like cell (*MUC*2^+^/*TFF*3^high^) signatures exist in both colon tumors and normal intestine. Furthermore, xenotransplantation of single CSC recapitulated the cell populations, immature progenitor and other epithelial cell populations, within a tumor. These findings suggest that differentiation of CSCs is a part of the mechanisms responsible for cancer cell heterogeneity within a colorectal tumor, in the same way as the differentiation of normal colorectal stem/progenitor cells creates heterogeneous cell populations in the epithelium.

### 2.2. Cell Surface Markers for Human Colorectal CSCs

#### 2.2.1. CD44 and Its Variants

CD44 has been used as a marker to isolate CSCs from various types of solid tumors such as breast [20], pancreatic [21], prostate [22] and colorectal cancers [5]. Xenograft tumors originated from EpCAM^+^/CD44^+^ colorectal cancer cells maintain a differentiated phenotype and are able to reproduce the full morphologic heterogeneity of their parental tissue [5]. Knockdown of CD44 suppresses colony formation and dramatically reduces tumor formation in xenografts [23].

CD44 is also expressed in stem/progenitor cells of variety of tissues such as central nervous system (CNS), lung, skin, liver, pancreas and intestine [24]. The intestinal stem cells are located at the bottom of intestinal crypt and responsible for the maintenance of the intestinal epithelium. Hierarchical structure of colorectal tissue is maintained by various niche factors, such as WNT, NOTCH, and SHH pathways. CD44 and LGR5 are the target genes of WNT signaling pathway [25] and mark CSCs and normal intestinal stem cells, suggesting that upregulation of WNT signaling pathways is important for both normal and cancer stem cells in the intestine.

**Figure 1:**
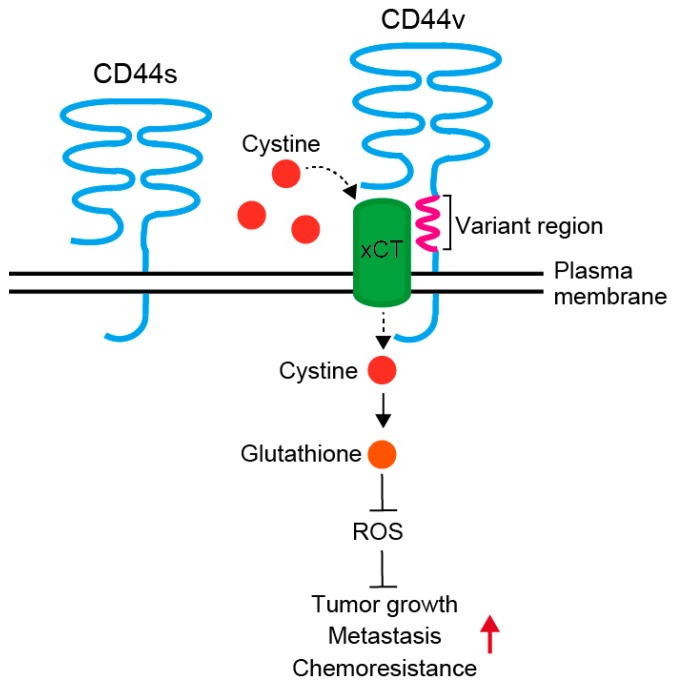
Regulation of oxidative stress by the CD44 variant isoform. Schematic illustration of a standard (CD44s) and variant (CD44v) isoforms of CD44. The variant region within CD44 molecule interacts with and stabilizes a subunit of a cystine/glutamate transporter (xCT), and promotes cystine uptake. Elevation of intracellular cystine level enhances the synthesis of antioxidant glutathione which detoxify reactive oxygen species (ROS), thereby promotes tumor growth, metastases and chemoresistance of CD44v expressing cells.

CD44 is a single chain transmembrane glycoprotein, also known as P-glycoprotein1, and involved in cell-cell and cell-matrix adhesion and cell signaling (Figure 1). Human CD44 gene consists of 20 exons and is located at chromosome 11 p. 13 [26]. CD44 has an extracellular domain, a transmembrane domain, and an intracellular domain [27,28]. The extracellular domain interacts with the exteRNAl microenvironment, and its main ligand is hyaluronic acid. In addition, it is able to interact with several other molecules, such as collagen, fibronectin, fibrinogen, osteopontin and matrix metalloproteinases [29]. Binding to these ligands is associated with various CD44 functions, such as lymphocyte homing, inflammation, angiogenesis, wound healing, cell migration and signaling [28].

The differential utilization of the 10 variant exons generates multiple CD44 variants (CD44v) with different combinations of variant exons. The predominant form of CD44 is translated to a polypeptide of 85–95 kD from mRNA composed of exons 1–5 and exons 16–20, and is designated hematopoietic or standard (CD44s) form [30]. Multiple variant isoforms (CD44v2–v10) arise from alternative splicing of the remaining 10 exons [31]. CD44v functions as promoter of tumor progression and metastases by reducing intracellular level of reactive oxygen species (Figure 1) [32,33]. Additional insertion in the membrane-proximal extracellular region of CD44v interacts with and stabilizes xCT, a subunit of the cystine-glutamate transporter, and promotes cystine uptake. This confers CD44v positive cells an ability to upregulate the synthesis of reduced glutathione to defend reactive oxygen species to drive tumor growth, metastasis and chemoresistance. At least, some of the CD44 variants are associated with cancer aggressive behavior, such as radioresistance, chemoresistance and metastases, and correlate with poor prognosis in a variety of human malignancies, including colorectal cancer [17,32,33,34,35].

#### 2.2.2. CD133

CD133 is a five-transmembrane glycoprotein and also known as Prominin-1 (PROM1). Human CD133 gene is located at chromosome 4 p. 15, consists of 36 exons, and is translated to a 120 kDa protein with 865 amino acids. This protein consists of a N-terminal extracellular domain, five transmembrane domains with two large extracellular loops and a 59 amino acid cytoplasmic tail [36]. Expression of CD133 is enhanced or suppressed by its intercellular binding molecule, HDAC6 or PTPRK, respectively [37,38].

CD133 is initially identified in CD34-positive hematopoietic stem cells [39,40], and then identified as a CSC marker in solid cancers, such as brain [41,42], prostate [22], liver [43], pancreas [44], lung [45], colon [4,6], and ovary [46]. CD133 expression is associated with poor prognosis in a number of tumor types. CD133 is also expressed in stem/progenitor cells of normal tissues, such as the bone marrow [40], brain [47], kidney [48], pancreas [49] and colon [50]. In the murine intestine, CD133-positive cells were localized at the bottom of the intestinal crypt and had the potential to differentiate into mature intestinal epithelial cells [50]. Activation of Wnt signaling pathway in Prom1 (CD133)^+/C−L^ mice resulted in a gross disruption of crypt architecture and a disproportionate expansion of Prom1-expressing cells at the crypt base [50], suggesting that CD133-expressing cells are origin of stem cells in the colorectal epithelium. Although CD133 is recognized as a stem cell marker, its biological function remains unknown. Neither ligands nor functions of CD133 and its variants are fully elucidated, and knockdown of CD133 in colon cancer cell lines does not influence the proliferation, migration, invasion, and colony forming abilities [51]. Splicing variants of CD133 are expressed tissue-specifically [52,53]; however, roles of each CD133 variants are not fully elucidated.

CD133 is the first marker proposed for the isolation of colon CSCs [4,6]. Analyses of primary colon cancers have revealed that CD133-positive colon cancer cells grow exponentially for more than one year in vitro as undifferentiated tumor spheres in serum-free medium, maintaining the ability to engraft and reproduce the same morphological and antigenic pattern of the original tumor [4]. In renal capsule xenograft models, CD133-positive human colon cancer cells are able to generate tumor, but CD133-negative cells are not [6]. These results show that CD133-positive colon cancer cells have stem cell properties, such as self-renewal, differentiation and high proliferation abilities.

However, there are contradictory data on the stem-cell-specificity and organ-specificity of CD133 expression [54]. Analyses of metastatic colon cancers have shown that both CD133-positive and CD133-negative colon cancer cells from metastatic lesions express CSC markers including CD44, and are able to form new tumors [55]. In addition, subcutaneous tumors derived from CD133-negative metastatic tumor cells grow faster than tumors derived from the CD133-positive cells. Therefore, CD133 is unlikely to be a marker of CSCs in a metastatic context [54,55]. It is reported that CD133 expression is not be limited to a stem cell population in the intestine [55], and posttranslational modification of CD133 changes as the CSCs differentiate [56]. The expression of AC133 epitope recognized by an anti-CD133/1 (AC133) antibody frequently used for colon CSC isolation decreases upon the differentiation of CSCs [56]. Therefore, contradictions may be caused due to the use of the antibodies for the detection of CD133: for example, anti-CD133/1 (AC133) and anti-CD133 (αhE2) antibodies have differential affinity to various glycosylated forms of CD133 [55]. Further research are required to clarify the difference between CSCs of primary tumors and those of metastatic tumors, and the effect posttranslational modification of CD133 on CSC identification.

#### 2.2.3. CD166

CD166, also known as activated leukocyte adhesion molecule (ALCAM), is a member of the immunoglobulin superfamily, originally identified by expression cloning of CD6 binding proteins, using COS cells transfected with c-DNA libraries [57]. CD166 expression is pathologically correlated with aggressive disease in a variety of cancers including melanoma [58], prostate [59], breast [60], ovarian [61] and esophageal cancers [62]. In human colorectal cancers, high expression of CD166 is strongly correlated with a shortened patient survival [63]. CD166 is a marker for CSCs in colon [5], lung [64] and prostate cancers [65]. Because CD44^+^/CD166^+^ double-positive cells are more tumorigenic than CD44^+^ single-positive cells in colorectal cancers, CD166 is an attractive marker for the further enrichment of CD44^+^ CSCs [5].

Human CD166 gene is located at chromosome 3q.13 and consists of 16 exons, and is translated to 105 kDa protein with 500 amino acids. This protein consists of five extracellular domains, a transmembrane domain and a short cytoplasmic domain. CD166 is involved in many biological functions, such as leukocyte stimulation and intravasation, monocyte migration, angiogenesis, hematopoiesis and cell-cell contact [57,66,67,68].

In the normal intestinal epithelium, CD166 is highly expressed in both crypt-based columnar cells (CBCs), intestinal stem cells, and Paneth cells at the crypt base of normal intestine [69]. A subset of CD166-positive CBCs co-express the stem cell markers, LGR5, Musashi-1(MSI1), or doublecortin-like kinase 1(DCLK1). In addition, the number of Lgr5^+^ stem cell is decreased and architecture of the intestinal epithelium is disrupted in the intestinal crypts of CD166^−/−^ mice [70]. These results suggest that CD166 is an important cell adhesion molecule for the formation and homeostasis of intestinal tissues.

#### 2.2.4. LGR5

LGR5, also known as G-protein coupled receptor 49 (GPR49) or 67 (GPR67), is a member of the G-protein coupled receptors. Lgr5^+^ colorectal cancer cells have higher tumorigenic and clonogenic abilities than Lgr5-negative colorectal cancer cells in xenotransplantation and/or organoid formation assays [71]. In the normal intestine, Lgr5 is expressed in cycling CBCs and single Lgr5^+^ colon stem cell has ability to generate an entire crypt when engrafted in the mouse colon [72].

Human LGR5 gene is located at chromosome 12q21, consists of 18 exons, and is translated to a 100 kDa protein with 907 amino acids. This protein consists of seven transmembrane domains and a large N-terminal extracellular domain that contains multiple leucine-rich repeats. LGR5 is a Wnt target gene and modulates Wnt signaling through binding its ligand R-spondin [73,74].

Recent two studies show that elimination of LGR5^+^ cells is dynamically compensated by the robust plasticity of other epithelial cells in both normal and malignant colon tissues [75,76]. Selective ablation of LGR5^+^ CSCs in LGR5-iCaspase9 knock-in organoids initially leads to tumor regression, but followed by tumor regrowth driven by re-emergence of LGR5^+^ CSCs from other cells [75]. Another study has shown that elimination of LGR5^+^ colon cancer cells prevents liver metastases, but does not inhibit primary tumor growth because of the plasticity of cancer cells within a primary tumor [76].

## 3. miRNAs for Stem Cell Regulation in the Human Colorectal CSCs

**Figure 2:**
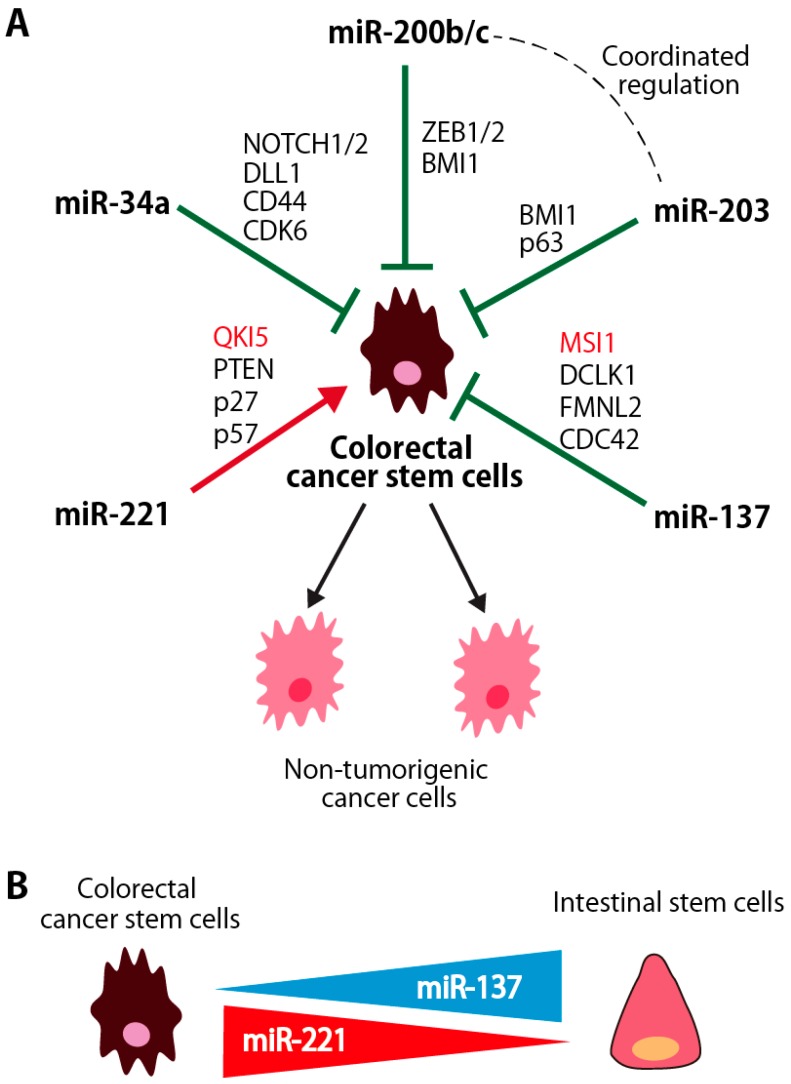
miRNAs involved in the regulation of colorectal CSCs. (**A**) Schematic illustration of the miRNAs that regulates colorectal CSC functions and their targets. miR-34a, miR-200b/c, miR-203 and miR-137 are downregulated in colorectal CSCs and suppress the stem cell properties of CSCs. Among them, the expression of miR-200bc and miR-203 is coordinately regulated, and these miRNAs coordinately function for the suppression of the stem cell properties of CSCs. In contrast, miR-221 is highly expressed in CSCs and enhances the stem cell properties of CSCs. These miRNAs target genes involved in the regulation of CSC properties. RNA-binding proteins shown in red font are described in detail in Chapter 4; (**B**) Differential expression of miRNAs between colorectal CSCs and normal intestinal stem cells. miR-221 and miR-137 are more highly expressed in colorectal CSCs and in normal intestinal stem cells than their counterpart, respectively.

miRNAs are non-coding RNAs with fewer than 25 nucleotides and regulate a variety of cell functions, including cell proliferation, stem cell maintenance and differentiation. Although the number of studies that analyzed the miRNA expression and/or functions in colorectal CSCs is still limited, miRNAs, such as miR-200 family miRNAs, miR-203, miR-137, miR-34a and miR-221, function as the regulator of stem cell properties in colorectal CSCs by targeting various genes involved in the regulation of CSC properties (Figure 2A). The roles of RBPs targeted by these miRNAs are described in Chapter 4. In addition, miRNAs, such as miR-137 and miR-221, are differentially expressed between CSCs and normal intestinal stem/progenitor cells, and will be candidate molecules to target CSCs in colorectal cancers (Figure 2B).

### 3.1. miR-200b/c and miR-203

#### 3.1.1. Coordinated Regulation of miR-200 and miR-203 in Colorectal CSCs

Recent study reports that some miRNAs will have a driving role in the establishment of CRC transcriptional subtypes [77]. miRNA master regulator analysis is applied to a paired mRNA–microRNA expression data set of 450 colorectal cancer samples, and 24 candidate miRNAs are identified. Functional validation in CRC cell lines has confirmed that miR-194, miR-200b, miR-203 and miR-429, which share target genes and pathways, have abilities to suppress the stem/serrated/mesenchymal subtype [77]. These results suggest that coordinated regulation of miRNAs will have a driving role in the establishment of the CRC transcriptional subtypes.

We recently identified a set of miRNAs that were coordinately regulated in the stem/progenitor cells in human normal intestinal epithelium. Among them, miR-200bc and miR-203 coordinately suppressed the stem cell properties of colorectal CSCs and normal intestinal stem/progenitor cells. These miRNAs regulate the genes involved in shaping the self-renewal and proliferative properties of stem cell populations, such as BMI1and ZEB1, and the expression of these miRNAs is negatively regulated by ZEB1. Therefore, the coordinated up-regulation of miRNAs with tumor-suppressor functions will be at least part of mechanism for the differentiation of human intestinal stem/progenitor cells and the suppression of colorectal CSCs.

#### 3.1.2. miR-200 Family miRNAs

The miR-200 cluster is an extensively studied tumor-suppressive miRNA cluster in the genome and composed of five miRNAs (miR-200a, miR-200b, miR-200c, miR-141 and miR-429). Based on the chromosomal locations, the miR-200 family is divided into two clusters: the miR-200ab/429 cluster is located on chromosome 1p36, and the miR-200c/141 cluster is located on chromosome 12p13 [78]. The miR-200 family members are also divided into two subgroups based on their seed sequences that differ by only 1 nt between the subgroups: miR-200b, miR-200c, and miR-429 (AAUACUG) and miR-200a and miR-141 (AACACUG). ZEB1 suppresses the expression of all miR-200 family members, which in turn inhibits the translation of ZEB1 mRNA, resulting in the double-negative ZEB1/miR-200 feedback loop [79]. In addition, super-enhancers, a new class of regulatory regions that consist of multiple enhancer-like elements, enhance both transcription and Drosha/DGCR8-mediated processing of primary miRNAs, including miR-200 family miRNAs [80].

A large number of studies demonstrated that strong suppressive effects of miR-200 family on cell transformation, cancer cell proliferation, migration, invasion, tumor growth and metastasis [81]. miR-200 family miRNAs function as a stem cell regulator through negative regulation of two key biological properties: EMT and self renewal [9,79,82]. The miR-200 family miRNAs downregulate ZEB1 and ZEB2 expression, and effectively upregulate the cellular E-cadherin level to maintain a cell in a more epithelial-like state. Furethermore, miR200c directly targets BMI1, a member of the Polycomb-group proteins required for the maintenance of multiple types of stem cells [9]. BMI1 inhibits apoptotic, senescence, and differentiation pathways by epigenetically repressing the transcription of Hox genes and the p16^Ink4a^ p19^Arf^ locus [78,83].

#### 3.1.3. miR-203

miR-203 located on human chromosome 14q32 is a putative tumor suppressor gene. RE1 silencing transcriptional factor (REST), EZH2 and SNAI1/2 are upstream transcriptional repressors of miR-203 [84,85,86,87].

miR-203 plays important role in stem cell regulation by targeting p63 and/or BMI1. p63 is an essential regulator of stem cell maintenance in the skin epithelium, and promotes epidermal differentiation [88]. Deletion of p63 in mice results in a dramatic loss of all keratinocytes and loss of stratified epithelia, probably due to a premature proliferative exhaustion of the stem and transient amplifying cells [89]. On the other hand, the EZH2-miR-203-BMI1 regulatory axis functions for the proliferation of neural stem/progenitor cells [87]. Overexpression of miR-203 suppresses the expression of BMI1 and colony formation in esophageal squamous cell carcinoma cell line [90]. In addition, miR-203 inhibits proliferation, migration, EMT and tumor angiogenesis in a variety of tumor cells by targeting SNAI2 [91,92], which in turn suppresses miR-203, forming a double negative feedback loop for the regulation of EMT [92].

#### 3.1.4. miR-137

miR-137 is located on human chromosome 1p21 and has been implicated to act as a tumor suppressor in colorectal and gastric cancers, glioblastoma and melanoma [93,94,95,96]. miR-137 negatively regulates the progression of colorectal cancer through directly targeting the oncogenes, such as MSI1, FMNL2 and CDC42 [97,98,99]. Epigenetic silencing of miR-137 by promoter hyper methylation contributes to early colorectal carcinogenesis [93].

miR-137 suppresses stem cell functions in various types of stem cells, such as neural stem cells and embryonic stem (ES) cells [100]. Recent study reported that miR-137 suppresses the expression of DCLK1, a microtubule-associated protein with C-terminal serine/threonine kinase domain. DCLK1 is highly expressed in colorectal CSCs, but not in normal intestinal stem/progenitor cells [101]. In contrast, miR-137 is much highly expressed in normal intestinal stem/progenitor cells than in colon CSCs (Figure 2B) [102]. In organoid growth assays, miR-137 specifically suppresses the development of organoids derived from colorectal CSCs through the inhibition of DCLK1 without affecting that of normal intestinal organoids, suggesting that miR-137 is a putative therapeutic target for colorectal CSC targeting therapy with less effect on the normal colorectal tissues.

#### 3.1.5. miR-221

miR-221 and miR-222 are encoded in tandem as a cluster on chromosome Xp11.3 and highly conserved in vertebrates [103]. miR-221 and miR-222 have the same seed sequence, and frequently function as oncogenes in human epithelial tumors. Overexpression of miR-221/222 is observed in human cancers such as colorectal [104], gastric [105], liver [106,107], pancreatic [108,109], and breast cancers [110], and glioblastoma [111]. miR-221 is frequently upregulated in human colorectal cancer tissues and the expression level of miR-221 was correlated with advanced TNM stage and the prognosis of colorectal cancer patients [104,112,113]. Oncogenic functions of miR-221 is partly mediated by its ability to promote cell cycle progression and drug resistance by targeting tumor suppressor genes, such as p 27, p 57 and PTEN [113,114].

Upregulation of miR-221 is observed in breast, pancreas and glioblastoma CSCs [9,115,116]. Recent paper reported that miR-221 and miR-200 family miRNAs play crucial opposing roles in inducing differentiation state in breast cancers [117]: miR-200 family miRNAs promote a well-differentiated epithelial phenotype, while miR-221/222 promote a poorly differentiated, mesenchymal-like phenotype. We found that expression of miR-221 was highly preferentially expressed in CSCs, but not in other non-tumorigenic cancer cells and normal intestinal epithelial cells, in human colorectal cancer patient specimens (Figure 2B). Then, we identified Quaking-5 (QKI-5), an RNA binding protein, as a novel target gene of miR-221 (Figure 2A). Knockdown of miR-221 suppressed the clonogenicity of colorectal CSCs in vitro in a QKI-expression dependent manner and an ability to form tumors in vivo. The tumor-suppressive function of QKI-5 is described in more detail in Section 4.3.

#### 3.1.6. miR-34a

miR-34 family is highly conserved in the evolutionary context. In vertebrates, miR-34 family consists of three members: miR-34a, miR-34b and miR-34c, which are generated from two distinct genomic loci. miR-34a is encoded by its own transcript located within the chromosome 1 p 36, while miR-34b and miR-34c are generated by processing a bicistronic transcript from chromosome 11q23. miR-34a is expressed at higher levels than miR-34b/c in most of the tissues including colorectal tissues, with the exception of the lung, where miR-34b/c is dominantly expressed [118].

miR-34a has a tumor suppressive role and suppresses many different oncogenic processes, such as differentiation, proliferation, migration and invasion [119,120,121]. miR-34a is a direct transcriptional target of p53 and component of its crucial tumor suppressor network [118,120,122,123]. p53 is inactivated in the most of colorectal cancers, and is posttranscriptionally induced by DNA damage and a number of additional cellular stresses [124]. miR-34a suppresses many oncogenes and stem cell regulators, including CD44, CDK6, c-Met, Notch-1, Notch-2, DLL1 and silent information regulator 1 (SIRT1) [119,125,126,127,128]. Bu and colleagues reported a unique miRNA-regulated mechanism in which miR-34a acts as a bimodal switch to target Notch in early-stage colon CSCs [129]; miR-34a directly targets Notch which promotes the self-renewal activity of colon CSCs, thereby regulating whether colon CSCs undergo symmetric or asymmetric division. Upregulation of miR-34a weakens Notch signaling and promotes the generation of daughter cells (non-CSCs), whereas low miR-34a level promotes Notch signaling and leads to the maintenance of CSCs. Analyses of Apc^Min/+^ mice carrying targeted deletions of the miR-34a and miR-34b/c genes showed that miR-34a/b/c suppress tumor formation caused by loss of Apc and modulate proliferation, apoptosis and tumor-associated immune defense [130].

Because of these broad anti-oncogenic function, miR-34 has been considered to be a novel therapeutic target, and MRX34, miR-34a mimics, becomes the first miRNA mimics to reach clinical trial for cancer therapy [131,132].

## 4. Roles of RNA Binding Proteins in Colorectal CSCs

**Figure 3:**
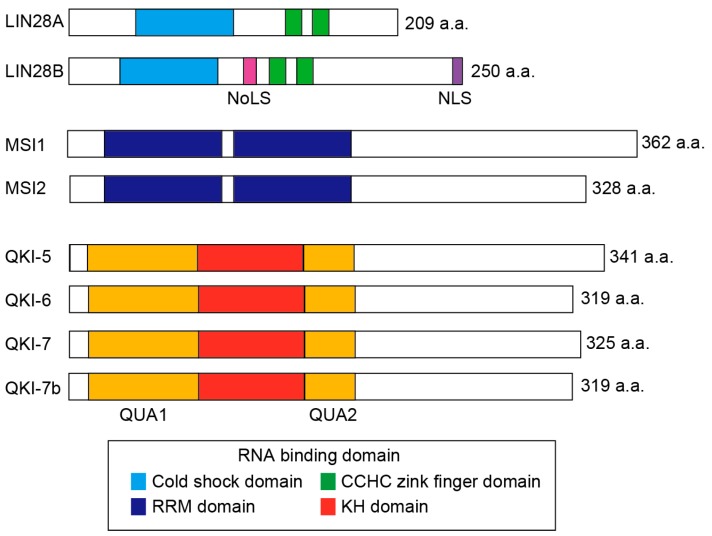
Domain structures of the RBPs. Schematics illustration of the three RBPs, LIN28A/B, MSI1/2, and QKI-5/6/7/7b. LIN28A/B have two types of the RNA-binding domains, a cold shock domain (CSD) and Cys-Cys-His-Cys (CCHC) zinc finger domains. MSI1/2 have two RNA recognition motif (RRM) domains. QKI-5/6/7/7b have an RNA binding domain, KH domain which is flanked by QUA1 and QUA2 regions. The structure of QKI proteins differ at their C-terminal. NLS, a nuclear localization signal; NoLS, a nucleolar localization signal.

RBPs are key components of RNA metabolism and functional dynamics. RBPs regulate a variety of RNA processing events, such as capping, splicing, cleavage, nucleotide editing, nuclear export, localization, stability, and translation [133]. There are more than 1500 RBPs in humans and some of them are evolutionary conserved [134]. RBPs manly interact with mRNAs via RNA-binding domains (RBDs), such as the cold shock, RNA recognition motif (RRM), heterogeneous nuclear RNP K-homology (KH), and zinc finger domains (Figure 3) [135].

RBPs have crucial roles in regulation of ES cells, and are involved in a number of human disease, such as neurological disorder and muscular atrophies [136]. However, their roles in cancer have not been fully elucidated. Here, we focus on LIN28 A/B, MSI-1/2 and QKI-5/6/7/7b, and describe their roles in the regulation of stem cells and CSCs.

### 4.1. LIN28A/B

#### 4.1.1. Molecular Characteristics

Lin28 is first identified in *C. elegans* through screens for lineage-modifying genes that alter developmental timing or heterochrony [137]. Lin28 functions by blocking the biogenesis of let-7 family miRNAs, and through direct translational enhancement or suppression of select messenger RNAs. Lin28 is an evolutionarily conserved RBP and has two forms, Lin28A and Lin28B, in humans and other mammals. Human *LIN28A* and *LIN28B* genes are located on chromosome 1p36 and 6q16.3, and encode proteins of 209- and 250-amino acids, respectively (Figure 3). Lin28 proteins have two RNA-binding motifs, a cold shock domain and Cys-Cys-His-Cys (CCHC) zinc finger domains [138]. In addition, LIN28B has a nuclear localization signal (NLS) and a nucleolar localization signal (NoLS). However, both LIN28A and LIN28B are mainly localized in cytoplasm.

#### 4.1.2. Roles in Stem Cell Regulation

LIN28 is highly expressed in ES cells, but is significantly downregulated in most of the differentiated adult tissues [139]. Lin28A null mice show early perinatal lethality, while Lin28B null mice show postnatal growth defects solely in males [140]. Double knockout causes embryonic lethality much earlier by E13, suggesting that these proteins have functional redundancy during development. Several studies showed that LIN28A/B are among the stem cell pluripotency factors. Using OCT4, SOX2, NANOG and LIN28A, adult human fibroblasts are successfully reprogrammed into induced pluripotent stem (iPS) cells [141]. Furthermore, OCT4, SOX2 and NANOG, three of the four Yamanaka factors, are able to activate LIN28A expression [142].

#### 4.1.3. Dysregulation in Cancer and CSCs

Increase of the expression level of LIN28 is associated with advanced human malignancies, such as breast, esophagus and colon cancers [143,144,145]. Recent studies revealed that LIN28A/B plays an important role in formation of CSCs, and contributes to tumor aggressiveness and metastasis [139,145,146]. Ovarian cancer cells co-expressing both LIN28A and OCT4 have a sub-population of cells with CSC properties [147]. In colon cancer, tumors with constitutive LIN28B expression exhibit increased expression of colorectal stem cell markers, LGR5, KIT and PROM1 (CD133) [145]. Recent study reported that Lin28B and its regulator IKKβ are able to maintain CSC properties via interaction with the WNT signaling pathway [148]. These findings suggest possible roles for LIN28B in intestinal CSCs. Because LIN28 is mainly expressed in the CSC population, but not in other non-tumorigenic cell population [145,149], and the LIN28/let-7 axis functions as a stem cell regulator, suppression of LIN28-let-7 interaction will be a potential strategy to target CSCs [150].

#### 4.1.4. Molecular Functions for the Regulation of Stem Cells and CSCs

LIN28 inhibits biogenesis of mammalian let-7 miRNAs, an important miRNA family consisting of 12 members located in genomic locations frequently deleted in human cancers [151], through direct binding to either pre-let-7 and/or pri-let-7 [152]. Activation of LIN28 occurs in several different primary human tumors, and these tumors display low levels of let-7 expression [153,154]. Downregulation of let-7 enhances the expression of its target genes, such as RAS, MYC and HMGA2, and promotes cancer initiation and progression [155,156,157]. Low expression of let-7 and high expression of LIN28 in non-small cell lung cancer patients was associated significantly with resistance to radiotherapy or chemotherapy [158]. LIN28 also exerts biological effects that are independent of let-7 miRNAs through selective binding to a large number of mRNAs. It is reported that LIN28 directly upregulates the expression levels of OCT4, IGF2, GPAA1, GNPDA1, HMGA1, EEF1G and RPS13 [159,160,161,162]. In addition, LIN28 phosphorylation by MAPK/ERK has little impact on let-7, but enhanced the effect of LIN28 on its direct mRNA targets, revealing a mechanism that uncouples the let-7-dependent and -independent activities of Lin28 [163].

### 4.2. MSI1/2

#### 4.2.1. Molecular Characteristics

*MSI* gene is identified as a regulator of asymmetric cell division of sensory organ precursor cells in Drosophila [164]. *MSI*1 and *MSI*2 genes are and evolutionarily conserved, and are located on chromosome 12q24 and 17q22 in human, respectively. MSI1 and MSI2 share about 75% amino acid identity. They belong to an RNA-binding protein family and contains two RNA recognition motif (RRM) domains, which recognize a defined sequence element within target mRNAs (Figure 3) [165,166].

#### 4.2.2. Roles in Stem Cell Regulation

MSI1 is mainly expressed in CNS stem cells and neural progenitor cells during development. MSI1 expression is detectable at the region where stem/progenitor cells are enriched, such as intestinal crypt, gastric gland, mammary epithelium and hair follicles [167,168,169,170,171]. MSI1 knockout mice die within two months after birth, because of a defect of CNS stem cells and a severe impairment of nervous system development [165,172].

MSI2 is more ubiquitously expressed in the tissues, including CNS [165,170]. MSI2 is a critical regulator of hematopoietic stem cells, where it has a function distinct from MSI1 [173]. MSI2 deletion leads to a decrease in hematopoietic stem cells, and its overexpression leads to an increase in hematopoietic stem/progenitor cells.

#### 4.2.3. Dysregulation in Cancer and CSCs

MSI1 expression has been reported in a variety of tumor cells [174], including colorectal [175,176], esophagus [177], bladder [178], lung [179], breast [180], cervical [181], and endometrial cancers [182], medulloblastoma [183], retinoblastoma [184], and glioblastoma [185]. MSI2 is highly expressed in leukemia and colorectal cancers [186,187]. In esophagus cancer, MSI1 expression is the highest in glandular structure during early cancer development, but it becomes weaker when adenocarcinoma progresses to the advanced stage [177], suggesting that MSI could be a marker of CSC during an early phase of tumor development.

Both MSI1 and MSI2 are frequently expressed in colorectal cancers. Higher expression level of MSI1 is correlated to the increased metastatic risk and poorer survival [175,176]. MSI2 is also central component in oncogenic pathway that promotes intestinal transformation [187]. Recent study shows that MSI1and MSI2 are functionally redundant in the colon, shares their binding target transcripts, and upregulate the PDK-Akt-mTORC1 axis [188]. Therefore, inhibition of both MSI1 and MSI2 are required to fully abrogate tumor growth in colorectal cancer cell lines.

#### 4.2.4. Molecular Functions for the Regulation of Stem Cells and CSCs

Current evidences suggest that MSI1 regulates stem cell functions in both normal and malignant colorectal cells, by transcriptionally suppressing genes with the tumor suppressor functions, such as Lrig1, Bmpr1a, Cdkn1a, Pten, p21, Numb and APC [187,189,190,191]. MSI1 translationally represses Numb by binding to its 3′ UTR of the mRNA, and upregulates Notch signaling that is important for a self-renewal activity [190].

### 4.3. QKI-5/6/7/7b

#### 4.3.1. Molecular Characteristics

*QKI* is evolutionarily conserved and located on chromosomes 6q26 in human. QKI is a member of the signal transduction and activation of RNA (STAR) protein family and composed of QUA1 and QUA2 regions, and a KH domain (Figure 3). There are at least four different mRNA splice variants in human: *QKI*-5, *QKI*-6, *QKI*-7, and *QKI*-7*b* [192,193]. QKI isoforms share exons 1–6, but differ in their C-terminal 35 amino acids encoded by exons 7 and 8 [192]. QKI-5 is the most abundant isoform in human colon and is predominantly nuclear, while QKI-6 can be nuclear and cytoplasmic, and QKI-7 is predominantly cytoplasmic [194]. QKI affects variety of RNA biogenesis, such as pre-mRNA splicing [195,196], mRNA stabilization [197], mRNA turnover [197], miRNA processing [198,199] and circular RNA biogensis [200].

#### 4.3.2. Roles in Stem Cell Maintenance and Differentiation

Several lines of evidence support that QKI will be a novel regulator of stem cell properties. QKI is an essential regulator of embryogenesis in both vertebrates and invertebrates [201,202]. QKI null mice were embryonic lethal at embryonic day 10.5 due to defective vascular remodeling and abnormal formation of vitelline vessels within the yolk sac [203]. QKI conditional knockout mice displayed severe hypomyelination in the CNS; tremors appears around postnatal day 10, and the mice dies by the third week after birth [204].

QKI plays important roles in the differentiation processes of endothelial cells (ECs) and erythrocytes. QKI-5 promotes EC differentiation from iPS cells by acting as a key regulator of CD144 stabilization and vascular endothelial growth factor receptor 2 (VEGFR2) activation through STAT3 signaling [205]. Human iPS cells overexpressing QKI-5 induces angiogenesis in Matrigel plug assays in vivo only seven days after subcutaneous injection into immunodeficient SCID mice. During erythropoiesis, QKI enhances or stabilizes the expression of miR-124 [199], which targets TAL1 and c-MYB, two transcription factors involved in normal erythropoiesis.

#### 4.3.3. Dysregulation in Cancer and CSCs

Expression of QKI is reduced by deletion or translocation in a number of human cancers, such as glioblastoma, and prostate, oral, gastric and colorectal cancers, and functions as a tumor suppressor [206,207,208,209,210,211,212]. Recent two large scale analyses reported that QKI is a major regulator of alternative splicing in lung cancer, and downregulation of QKI protein is an independent factor for poor prognosis [213,214]. In glioblastoma, QKI suppresses TGFβ signaling pathway by upregulating miR-20a, and impairs the stem cell functions of glioma stem cells [206,215]. Furthermore, MYB-QKI fusions is a candidate driver event in angiocentric gliomas [207]. MYB-QKI rearrangements promote tumorigenesis through three mechanisms: MYB activation by truncation, enhancer translocation that drives aberrant MYB-QKI expression, and hemizygous loss of a tumor suppressor gene, QKI.

#### 4.3.4. Molecular Functions for the Regulation of Stem Cells and CSCs

QKI epigenetically regulates the expression of multiple genes involved in stem cell maintenance and differentiation, such as SOX2, NANOG and OCT4 [208,215]. QKI impairs the stem cell functions of glioma stem cells [206,215], and neural stem cells [215]. *QKI* deletion on a *Pten*^−/−^; *Trp*53^−/−^ background enhances the maintenance of neural stem cell function and results in glioblastoma formation with a penetrance of 92%.

Several studies reported that QKI is the mRNA splicing regulator [213,216]. QKI-5 repressed the inclusion of NUMB alternative exon through competing with a core splicing factor SF1 [213]. AlteRNAtive pre-mRNA splicing, the process by which multiple mRNA variants can be produced from a single gene, is a key mechanism for increasing proteomic diversity [217]; and is important for oncogenesis, and CSC regulation. For example, splicing variants of a stem cell marker CD44 functions as promoter of tumor progression and metastases at least partly by reducing intracellular level of reactive oxygen species (Figure 1) [32,33,35], suggesting that the regulation of splicing will play an important role in the regulation of the CSC functions. In addition, STAR protein family proteins, which QKI belongs to, are involved in the splicing regulation, including that of CD44 [218]. Although, further studies are required to reveal roles of alternative pre-mRNA splicing in the regulation of CSCs, QKI will be a one of the epigenetic regulator of CSC functions involved in this process.

## 5. Conclusions

Cancer has been mainly viewed as a disease driven by the accumulation of genetic mutations. However, in the past decade, many studies also reported the importance of epigenetic regulation in cancer initiation, progression, metastases and therapy resistance. Epigenetic mechanisms help establish cellular identities, and failure of these processes can result in cancer formation by inappropriately activating or inhibiting various signaling pathways [219]. 

We summarized the function of two important epigenetic regulators, miRNAs and RBPs, in regulation of colorectal CSCs. It is possible that the functional interactions between miRNAs and RBPs, such as miR-221 and QKI, is one of the key networks in CSC biology. Further analyses of the expression and roles of miRNAs and RBPs in colorectal CSCs will promote our understanding of the molecular mechanism for the regulation of CSCs, and propose biomarkers and therapeutic targets for CSCs in colorectal cancers.
